# Note from the editors: Acute hepatitis among children, establishing evidence and baselines for comparison

**DOI:** 10.2807/1560-7917.ES.2022.27.19.220512m

**Published:** 2022-05-12

**Authors:** Eurosurveillance editorial team

**Affiliations:** 1 European Centre for Disease Prevention and Control (ECDC), Stockholm, Sweden

**Keywords:** hepatitis, outbreak, baseline

Two rapid communications in this issue report on attempts to gauge the extent of the recently reported increase in acute hepatitis cases among children in several countries in Europe and on other continents. They show that at the start of a possible outbreak with a yet unknown aetiology but features suggestive of an infectious cause, it is challenging to establish a sound evidence-base and to measure increase against a baseline of a syndrome or disease which is not under systematic surveillance and governed by standard case definitions. The short communications by van Beek et al. [1] and de Kleine et al. [2] both report results on quick surveys among different networks of clinical experts working with (paediatric) liver diseases and use different approaches to try and shed light on whether there is—or is not—a widespread increase. Both cover a similar period in 2022 and apply a similar case definition for acute hepatitis of unknown aetiology.

Van Beek et al. set out to establish the incidence for acute hepatitis of unknown aetiology in children over the previous 5 years (2017─21) as comparison baseline to assess the increase and included data from the United Kingdom (UK). They further investigate geographical distribution of cases up to 26 April 2022. This year, of 24 countries with experts responding, five of 17 identified an increase in probable cases of unexplained acute hepatitis in European countries and in one of seven non-European countries. An increase in severe cases was noted in five European countries.

De Kleine et al. establish estimates of paediatric acute liver failure (pALF) registered by specialised liver centres within the European Reference Network on Hepatological Diseases over 3 previous years (2019─21) that fulfil the definition of the Paediatric Acute Liver Failure Study Group [3]. The survey also captures the number of paediatric hepatitis cases and pALF at the centres in the first 3.8 months of 2022. The 34 participating centres in Europe and Israel, not including the UK, detected a mean of 2 (range: 0–8) up to 26 April 2022. Twelve reported a suspected increase but documented no rise in numbers, and 22 reported no suspicion of an increase. Of 64 children with severe hepatitis and/or pALF with further details available, a cause was identified in 11, while for 27 it remained indeterminate and for 26 unknown.

The results from these surveys show that the condition remains rare also in 2022. They do not provide a uniform picture but indicate that while in some countries or centres there are signals for an increase in cases, this is not evident in others. As examples of the former, Marsh et al. described the clear signal seen in Scotland and the initial investigation and clinical details of the first Scottish cases on 14 April [4] and the UK Health Security Agency published a technical briefing on 25 April [5].

Despite acknowledged limitations, the two Rapid communications in this issue provide some useful information in the form of ‘best available evidence’ at this moment in time. International collaboration is needed to support robust and timely epidemiological and clinical investigations to establish if there is an excess of cases of severe hepatitis of unknown origin that demands targeted studies to determine the aetiological agent. Over the coming weeks we hope to see more data and results from ongoing investigations that should help to fill the present gaps.
